# The Association of On-Admission Blood Hemoglobin, C-Reactive Protein, and Serum Creatinine With 2-Year Mortality of Patients With Femoral Neck Fractures

**DOI:** 10.1177/21514593211037758

**Published:** 2021-08-18

**Authors:** Arkan Sayed-Noor, Bariq Al-Amiry, Alan Alwan, Björn Knutsson, Björn Barenius

**Affiliations:** 1 Department of Clinical Science and Education, Södersjukhuset, 27106 Karolinska Institute, Stockholm, Sweden; 2 Department of Clinical Science, Intervention and Technology, 206106 Karolinska Institute, Stockholm, Sweden; 3 Department of Surgical and Perioperative Sciences, 59588 Umeå University, Umeå, Sweden

**Keywords:** hip fracture, femoral neck fracture, mortality, anemia, C-reactive protein, creatinine

## Abstract

**Introduction:**

The mortality of femoral neck fracture (FNF) is high and every effort should be made to identify and manage any possible risk factors. The aim of this study was to evaluate whether on-admission hemoglobin (Hb) level, C-reactive protein (CRP), and serum creatinine were associated with 2-year mortality after FNF.

**Patients and Methods:**

In this retrospective observational cohort study, we considered for inclusion all displaced FNF patients 65 years and above treated with hemi-arthroplasty between February 2011 and May 2015. We documented the age, sex, cognitive status, and American Society of Anesthesiologists (ASA) classification. The Hb level, CRP, and serum creatinine were measured. The medical records were followed up for 2 years. We fitted different crude and adjusted Cox proportional hazards models to examine whether Hb level <100 g/L, CRP >20 mg/L, and serum creatinine >100 μmol/L were associated with the 2-year mortality, adjusted for age, sex, and ASA class.

**Results:**

A total of 290 patients [208 females (72%), mean age 84 years] were included in the study. More than 50% of patients had impaired cognition and ASA class 3–4. Of the 290 patients, 38.3% (*n* = 111) had died within 2 years after surgery. Mortality among males was 46.3% (*n* = 38) while mortality among females was 35,1% (*n* = 73), *p* = 0.07. We found that on-admission Hb level <100 g/L was associated with 2-year mortality (HR = 3.3, 95% CI: 1.3–8.3, *p* < 0.01) while CRP >20 mg/L and serum creatinine >100 μmol/L were not associated with 2-year mortality (*p* = 0.89 and *p* = 0.31, respectively).

**Conclusion:**

On-admission Hb level <100 g/L, but not CRP >20 mg/L and serum creatinine >100 μmol/L, was associated with 2-year mortality. These results can help healthcare providers identify high-risk FNF patients who probably would benefit from optimized perioperative medical management.

## Introduction

Hip fractures are common devastating injuries affecting elderly people, usually after a fall from a standing position. Nearly half of these injuries are femoral neck fractures (FNFs). Approximately 320,000 hip fractures occurred each year in the United States, 76,000 in the United Kingdom, and 16,000 in Sweden.^1-3^ The number of these injuries is expected to rise owing to an increased aging population, exposing the community and healthcare to a challenging burden. Many of these individuals have medical comorbidities such as cardiovascular, pulmonary, and renal diseases as well as osteoporosis and balance disorders with a falling tendency. Accordingly, the perioperative mortality is high, and those who survive suffer from deteriorating general health and walking ability.^4^ Every effort should therefore be made to detect and manage any factors that are associated with the increased mortality. Previous studies have shown that old age, male gender, the presence of medical comorbidities and cognitive impairment, low caseload, and delay of more than 48 h of surgical treatment are some important risk factors.^5-7^ Furthermore, certain routine blood tests could also reflect the health status and were associated with mortality after FNF. For instance, low Hb, low albumin, and high creatinine on admission have been reported to be associated with increased mortality.^8-10^ However, the majority of these reports included mortality rates for the first postoperative year only.

The purpose of this observational cohort study was to determine whether on-admission Hb level, CRP, and serum creatinine were associated with the 2-year mortality after FNF. We hypothesized that Hb level <100 g/L, CRP >20 mg/L, and serum creatinine >100 μmol/L were associated with 2-year mortality.

## Patients and Methods

### Study Setting

This is a retrospective cohort study that was conducted at Sundsvall Teaching Hospital, Sundsvall, Sweden. This hospital is a secondary referral emergency center affiliated with Umeå University with a catchment area of approximately 160,000 inhabitants.

### Study Subjects and Data Collection

Between February 2011 and May 2015, 290 patients aged ≥65 years with Garden classification III and IV, that is, displaced FNF, were admitted to the orthopedic department. None of them was non-walker, non-Swedish speaker, or had degenerative joint disease or rheumatoid arthritis of the fractured hip. At admission, clinical data regarding patient demographics, medical comorbidities [American Society of Anesthesiologists (ASA) classification], as well as routine blood tests, including blood hemoglobin (g/l), CRP (mg/l), and serum creatinine (umol/l), were documented. Furthermore, patients’ cognitive status was assessed for the last week before the fracture, in a retrospective rating using the short portable mental status questionnaire (SPMSQ),^11^ as follows: 0–2 severe, 3–5 moderate, 6–7 mild, and 8–10 no cognitive impairment. In patients with SPMSQ score of less than 6, all clinical variables except cognitive status were assessed by means of a report from a close relative or nursing home staff as described and used by Blomfeldt et al.^12^ According to the SPMSQ score, we divided the cohort into two groups. The group of patients with SPMSQ score of less than 6 was defined as cognitively impaired, while the group of patients with SPMSQ score of 6 or more was defined as lucid.

After adequate preparation for surgery, all patients were operated with hemi-arthroplasty within 48 h from admission. The cemented Lubinus SPII stem with unipolar head (Link^®^, Germany) was used. Postoperatively, patients were mobilized and rehabilitated by physiotherapists according to the patients’ physical and mental status. The patient was then discharged to suitable accommodation.

By using the unique Swedish personal ID number, we collected data and documented the postoperative mortality up to 2 years by consulting the electronic patient files.

### Statistical Analysis

Parametric data were presented as means and standard deviations (SDs) while categorical data were presented as counts and percentages. We applied crude and adjusted Cox proportional hazards models to test our hypotheses of whether on-admission Hb levels <100 g/L, CRP >20 mg/L, and serum creatinine >100 μmol/L were associated with the 2-year mortality. In a post hoc approach, we used the method of Peduzzi et al. to estimate whether the study sample size was adequate.^13^ They found that at least ten events per variable were required to get an acceptable regression coefficient. As the number of patients with Hb <10 g/L, CRP >20 mg/L, and serum creatinine >100 μmol/L exceeded 30 patients in each group, a total of three variables could be included in the regression analysis. We included priori confounding factors of age, gender, and ASA class. We chose these factors as we anticipated them to be related both to exposure and outcome, and that they would not be in the causal pathway. Hazard ratios (HRs) and 95% confidence intervals (CIs) were presented. A *p*-value < 0.05 was considered significant. All analyses were performed using SPSS statistical software version 22.

### Ethics

The study was conducted in accordance with the ethical principles of the Declaration of Helsinki. The Ethics Committee at Umeå University approved the study (Dnr. 2011-428-31M).

## Results

The study group consisted of 290 patients. Their demographic data are presented in Table 1.

**Table 1.** The Demographics of the Study Population.

Study group	*n*= 290 patients
Age	Mean = 84 years
	SD = 6
	Range = 61–90 years
Sex	208 females (72%)
	82 males (28%)
Cognitive status measured by SPMSQ	140 patients (48%) were lucid
	150 patients (52%) with cognitive impairment
ASA class	130 patients (45%) with ASA 1–2
	160 patients (55%) with ASA 3–4

SD, standard deviation; SPMSQ, the short portable mental status questionnaire; ASA class, American Society of Anesthesiologists classification.

The Hb level <100 g/L was found in 35 patients (12%), CRP >20 mg/L in 91 patients (31%), and serum creatinine >100 μmol/L in 71 patients (24%).

The 2-year mortality was 38.3% [*n* = 111, 73 females (35% of female patients) and 38 males (46% of male patients), *p* = 0.07].

**Figure 1.**
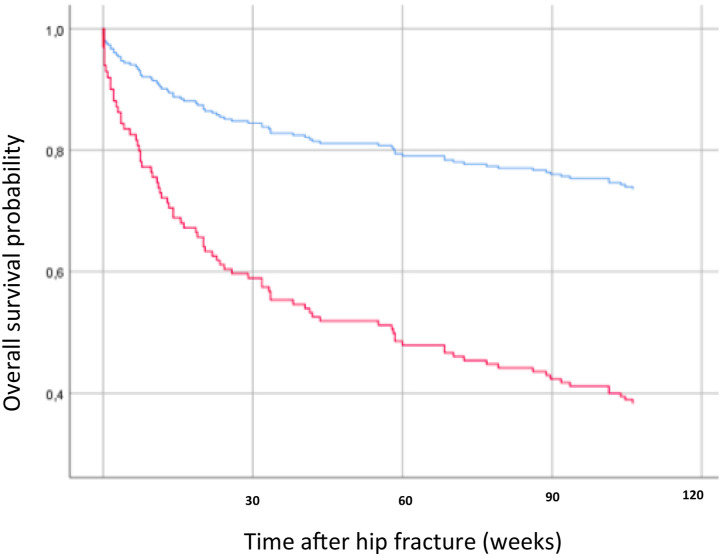
Kaplan–Meier curves displaying the estimated survival probability for FNF patients with on-admission Hb level <100 g/L [the below curve (red) line] compared to Hb level ≥100 g/L [the above curve (blue) line], adjusted for age, sex, and ASA class. FNF, femoral neck fracture; ASA, American Society of Anesthesiologist.

The crude model of Cox proportional hazards analysis showed that Hb level <100 g/L was associated with the 2-year mortality. When the model was adjusted for age, sex, and ASA class, Hb level <100 g/L was still associated with 2-year mortality (HR = 3.3, 95% CI: 1.3–8.3, *p* < 0.010) (Table 2). Figure 1 displays the Kaplan–Meier curve for the adjusted model.

**Table 2.** Hazard Ratios With 95% Confidence Intervals for the 2-Year Mortality.

	Crude model	*p*-value	Adjusted model*	*p*-value
Hb				
≥100 ref	1			
<100	3.1 (1.3–7.7)	0.014	3.3 (1.3–8.3)	<0.01
CRP				
≤20 g/L ref	1			
>20 g/L	1.0 (0.99–1.01)	0.24	1.0 (0.99–1.01)	0.89
Crea				
≤100 μmol/l ref	1			
>100 μmol/l	1.4 (0.9–2.3)	0.15	1.3 (0.78–2.1)	0.31
Cognitive status				
Lucid ref	1			
Impaired	2.4 (1.5–3.9)	<0.01	2.3 (1.4–3.8)	<0.01

Hb, hemoglobin (Hb); CRP, C-reactive protein; Crea, serum creatinine; Ref, reference.

^*^ Adjusted for age, sex, and ASA class.

The crude model of Cox proportional hazards analysis showed that CRP >20 mg/L was not associated with the 2-year mortality (HR = 1.0, 95% CI: 0.99–1.01, *p* = 0.24). When the model was adjusted for age, sex, and ASA class, CRP >20 mg/L was still not associated with 2-year mortality (HR = 1.0, 95% CI: 0.99–1.01, *p* = 0.89) (Table 2).

Regarding serum creatinine, the crude model of Cox proportional hazards analysis showed that serum creatinine >100 μmol/L was not associated with 2-year mortality (HR = 1.4, 95% CI: 0.9–2.3, *p* = 0.15). When the model was adjusted for age, sex, and ASA class, serum creatinine >100 μmol/L was still not associated with 2-year mortality (HR = 1.3, 95% CI: 0.8–2.1, *p* = 0.31) (Table 2).

Cognitive impairment was associated with the 2-year mortality both in the crude (HR = 2.4, 95% CI: 1.5–3.9, *p* < 0.01) and adjusted analysis (HR = 2.3, 95% CI: 1.4–3.8, *p* = 0.001) (Table 2).

## Discussion

The results of this study show that in patients with displaced FNF treated with hemi-arthroplasty, on-admission Hb level <100 g/L, but not CRP >20 mg/L or serum creatinine >100 μmol/L, was associated with 2-year mortality. Also, impaired cognition was associated with 2-year mortality. These results indicate the prognostic importance of on-admission Hb level on short to mid-term mortality.

Several publications have discussed the value of on-admission blood tests on early and late complication and mortality rates in hip fracture patients.^8-10,14-19^ They aimed to use these tests, either alone or in combination, as predictive factors. However, the majority of these studies discussed the first postoperative year where the short-term complication and mortality rates are relatively high and hence of interest. In the present study, we investigated the results at 2-year follow-up since these results are sparse in the literature and further studies are therefore warranted.

Anemia is a common health issue in elderly people. While mild anemia has not been found to increase long-term mortality, moderate to severe anemia according to the World Health Organization’s definition has been associated with other comorbidities, declined physical activities (including gait and balance difficulties), and increased mortality.^20^ The definition and incidence of anemia in hip fracture patients have varied among studies where the Hb cut-off value commonly ranged from 100 up to 130 g/L.^8,14-15^ Most of these studies, as in the present one, have dichotomized Hb values, instead of using it as a continuous variable, and made no adjustment for gender differences. This might allow easier comparison of their results.

We had 35 patients (12%) with Hb <100 g/L, an incidence that is comparable to other reports. Laulund et al.^21^ found that low Hb levels were correlated with higher mortality. In their review, they calculated a pooled odds ratio of 2.78 with 95% CI: 2.17–3.55, *p* < 0.001. The seven studies included variable follow-up length and Hb cut-off values. On the other hand, Potter et al.^22^ reported that anemia on admission was associated with greater risk of 30-day, 90-day, and 1-year mortality. In their review, the pooled relative risk was 1.64, 95% CI: 1.47–1.82, *p* < 0.001. After adjustment for co‐morbidities, the association of anemia with increased mortality remained in four of eight observational studies. Similar to our study, Zhang et al.^23^ studied the risk of all-cause mortality at 2 years after hip fracture and found that age, female sex, and ASA score were associated with on-admission anemia. In their study, Cox proportional hazards regression analysis suggested that the risk of all-cause mortality was higher in the anemia group on admission (HR = 1.680, 95% CI: 1.201–2.350, *p* < 0.01). Furthermore, their take-home message was when anemia is used for mortality prediction in these patients, a specific time point should be chosen. They suggested that only on-admission anemia should be used for mortality prediction, but not postoperative or discharge anemia. On the other hand, Carson et al. performed the Transfusion Trigger Trial for Functional Outcomes in Cardiovascular Patients Undergoing Surgical Hip Fracture Repair (FOCUS) to test the whether a higher threshold for blood transfusion with a median of two units of red blood cells at a hemoglobin level of 100 g/L would improve functional recovery and reduce morbidity and mortality, as compared with a more restrictive transfusion strategy (a hemoglobin level of <80 g/L or symptoms).^24^ They found that the liberal transfusion strategy did not reduce rates of death or inability to walk independently on 60-day follow-up or reduce in-hospital morbidity in elderly patients at high cardiovascular risk.^24^

The CRP, beside other parameters, has been used to evaluate the perioperative inflammatory response in hip fracture patients.^9,18,25^ However, its clinical value as prognostic factor for short- and long-term mortality is still debatable. As with Hb levels, the cut-off value of CRP used in different studies is widely variable.^8,18,25^ For instance, Kim et al.^18^ found preoperative CRP >10 mg/L to be an independent predictor for 1-year mortality with an odds ratio of 2.04, 95% CI: 1.09–3.80, *p* = 0.025. Also Fakler et al.^26^ showed that patients with a mild CRP (10–39.9 mg/L) or active inflammatory response (CRP  ≥ 40 mg/L) had a higher 1-year mortality of 33 and 40% respectively, compared to 16% in patients with no (CRP  < 10 mg/L) inflammatory response (*p* = 0.002). On the other hand, Capkin et al.^9^ investigated the role of CRP (mg/l)/albumin (g/dl) ratio in 1-year mortality and found that an increased ratio above 2.49 increased the mortality, while Ren et al.^27^ described another ratio of CRP/Prognostic Nutritional Index and found prognostic for 1-year mortality. In contrary to these studies, we found no association between CRP > 20 mg/L and 2-year mortality. This could be due to differences in cut-off values and result calculations among studies. Another explanation could be that the eventual association between CRP and mortality during the first postoperative year fades out when examined at 2-year follow-up.

The third blood test in our study was serum creatinine (umol/l), which is a commonly used maker for renal function. Glomerular filtration rate is another, maybe more reliable, test to reflect the renal function. Patients with advanced renal disease have a well-established increased risk of both sustaining hip fractures and postoperative mortality.^10,14,19,28^ Also, patients with post-hip fracture acute renal injury, revealed by increased serum creatinine, showed a higher mortality rate up to 4 years postoperatively compared with patients with normal renal function (HR = 1.63, 95% CI: 1.14–2.31; *p* = 0.007).^10^ In contrast to our results, several other studies have shown that pathologically high serum creatinine increased short- and long-term mortality in hip fracture patients.^10,14,19,28^ This discrepancy, as with the CRP results, could be due to the differences among the studies’ materials in timing blood tests, using cut-off values, and perioperative management of patients with high versus normal serum creatinine.

### Limitations

This study has some limitations. First, as an observational, retrospective study, there was an inherent design limitation. Patients with missed values of blood tests were excluded. The included blood tests were taken on admission where patients could have different hydration and nutritional status. This could influence accuracy of the results. The values of the blood test were dichotomized instead of being used as continuous. This might be a less accurate way to evaluate the degree of severity of these blood tests on the mortality. However, most of the published reports have used this approach and we chose the same method in order to be able to compare our results to others. Unfortunately, the discrepancy in using different cut-off values among studies is a disadvantage that jeopardizes the comparison. Another limitation is the residual confounding of factors that might have affected the mortality and were not included in regression models.

## Conclusion

On-admission Hb level <100 g/L, but not CRP >20 mg/L and serum creatinine >100 μmol/L, was associated with 2-year mortality. These results can help healthcare providers identify high-risk FNF patients who probably would benefit from optimized perioperative medical management.
